# Ultrasensitive loop mediated isothermal amplification (US-LAMP) to detect malaria for elimination

**DOI:** 10.1186/s12936-019-2979-4

**Published:** 2019-10-16

**Authors:** Abu Naser Mohon, Sisay Getie, Nusrat Jahan, Mohammad Shafiul Alam, Dylan R. Pillai

**Affiliations:** 1 Department of Microbiology and Infectious Disease, Cumming School of Medicine, University of Calgary, Alberta, T2N 4N1 Canada; 2 Department of Pathology and Laboratory Medicine, Cumming School of Medicine, University of Calgary, 9-3535 Research Road NW, 1 W-416, Calgary, AB T2L2K8 Canada; 3 Department of Medicine, Cumming School of Medicine, University of Calgary, Alberta, T2N 4N1 Canada; 4 Department of Medical Parasitology, School of Biomedical and Laboratory Science, College of Medicine and Health Sciences, University of Gondar, P. O. Box: 196, Gondar, Ethiopia; 5 Emerging Infections and Parasitology Laboratory, International Center for Diarrheal Disease Research, Bangladesh (icddr,b), Dhaka, 1212 Bangladesh

**Keywords:** Malaria, Diagnostics, LAMP, Ultrasensitive, Elimination, Method, Asymptomatic

## Abstract

**Background:**

Malaria elimination requires diagnostic methods able to detect parasite levels well below what is currently possible with microscopy and rapid diagnostic tests. This is particularly true in surveillance of malaria at the population level that includes so-called “asymptomatic” individuals.

**Methods:**

The development of the first ultrasensitive loop mediated amplification method capable of detecting malaria from both whole blood and dried blood spots (DBS) is described. The 18S rRNA and corresponding genes that remain stable on DBS for up to 5 months are targeted.

**Results:**

In the case of *Plasmodium falciparum*, lower limits of detection of 25 parasite/mL and 50–100 parasite/mL from whole blood and DBS were obtained, respectively. A sensitivity of 97.0% (95% CI 82.5–99.8) and specificity of 99.1% (95% CI 97.6–99.7) was obtained for the detection of all species in asymptomatic individuals from Africa and Asia (n = 494).

**Conclusion:**

This tool is ideally suited for low middle-income countries where malaria is endemic and ultrasensitive surveillance of malaria is highly desirable for elimination.

## Background

In 2016, there were approximately 216 million estimated cases of malaria identified globally [1]. However, more cases remained undetected as the diagnosis of low-level infections is challenging at the field level. Approximately, 20–70% of the malaria infections are reported to be undetected by current non-nucleic acid tests such as microscopy and rapid diagnostic tests (RDTs) [2]. RDTs and microscopy can detect *Plasmodium* infection when the parasite count is higher than 50,000–200,000/mL of whole blood [3–5]. Molecular detection tools have provided a window into these undetected cases which comprise the asymptomatic reservoir from which transmission can occur [6]. Questions remain as to whether these individuals are truly asymptomatic, but it is becoming apparent that they contribute to onward transmission [7–9]. Low-level infections can be detected by molecular diagnostic tools, such as PCR, real time PCR (qPCR) and reverse transcriptase-qPCR (qRT-PCR); however, these methods are time-consuming, require expensive instruments for initial laboratory set up as well as expert personnel to operate the laboratories [10–12].

In contrast to PCR or qPCR, loop-mediated isothermal amplification (LAMP) based methods are quick, simple, and require little capital equipment. Several in-house LAMP assays have efficiently detected *Plasmodium* infection [13, 14] as well as identified drug resistance associated genetic markers in *Plasmodium falciparum* [15, 16].

Currently, two commercial kits are available in the market: LoopAmp malaria (Pan/Pf) detection kit (Eiken Chemical Company, Tokyo, Japan) and *Illumigene* malaria LAMP assay (Meridian Biosciences, Cincinnati, USA), both of them have been reported to detect symptomatic malaria cases with high sensitivity and specificity [10–12, 17–19]. These kits possess a limit of detection (LOD) of approximately 1000 parasites/mL; however, at the field level, parasite density can be much lower than 1000 parasites/mL. In some instances, almost 50% of the asymptomatic patients harbour a parasite level that beyond the LOD of these two kits (i.e. < 1000 parasite/mL) [6, 20]. For example, the LoopAmp malaria kit was reported to be only 40.8% sensitive in an asymptomatic malaria survey in Zanzibar [20]. Therefore, the efficiency of the commercial LAMP assays are equivalent to the gold standard nested PCR which has a LOD ranging from 1000 to 10,000 parasites/mL [21]. However, 18S rDNA based high volume qPCR [22] and 18S rRNA based qRT-PCR [23] have been reported recently to have a LOD of approximately 20 parasites/mL from whole blood. Additionally, 18S rRNA was found to be stable in filter paper dried blood spots (DBS) for up to 6 months while providing enough template to be detected by qRT-PCR [24]. Previously, a field-tailored reverse transcriptase LAMP assay demonstrated a LOD of 0.8 parasite/mL of whole blood using the transcript of gene *exp1* [25]. Similarly, 18S rRNA is known to be a stable target for qRT-PCR and advantageous as a multicopy target because a single *P. falciparum* parasite contains approximately 10,000 copies of 18S rRNA at the ring stage [23]. Although such quantitative data was not obtained for other species, some copies of the 18S rRNA gene are highly expressed at the asexual blood stages in other species [26–28].

Here, a single reaction tube, low-cost ultrasensitive LAMP (US-LAMP) test targeting 18S rRNA for all malaria species was developed. This relatively inexpensive and easy-to-perform test works from whole blood and dried blood spots (DBS) and can be used for ultrasensitive visual detection of malaria for elimination surveillance efforts in low middle-income countries (LMIC).

## Methods

### Primer design

For *Plasmodium* genus-level detection, a set of genus-specific (Pan) primers was adopted from a previous study [13]. Modified *P. falciparum*-specific primers (Pf) were developed for this study (Table 1) [29]. Briefly, *P. falciparum*-specific primers were modified to amplify a specific region of the 18S rRNA gene located at chromosome 5 and 7, since these two copies are highly expressed at the blood stages of their life cycle [23, 30].

**Table 1:** List of primers used in this study

Primer sets	Primer name	Sequence
Genus (Pan-Lamp) [17]	F3	GTATCAATCGAGTTTCTGACC
	B3	CTTGTCACTACCTCTCTTCT
	F1P	TCGAACTCTAATTCCCCGTTACCTATCAGCTTTTGATGTTAGGGT
	B1P	CGGAGAGGGAGCCTGAGAAATAGAATTGGGTAATTTACGCG
	LPF	CGTCATAGCCATGTTAGGCC
	LPB	AGCTACCACATCTAAGGAAGGCAG
*P. falciparum* (Pf-LAMP) (modified from [29])	F3	TGGTGG GAATTTAAAACCTTC
	B3	CGCTTTAATACGCTT CCT C
	F1P	GCTATTGGAGCTGGAATTACCGCAGAGTAACAATTGGAGGG
	B1P	GTTGCAGTTAAAACGCTCGTAGTCTAAAATAGTTCCCCTAGAATAGT
	LPF	CTGCTGGCACCAGACTT
	LPB	TGAATTTCAAAGAATCGATATTTTATTGTAACT

### Nucleic acid extraction-whole blood

For whole blood specimens, a modification of the traditional Trizol Reagent (Invitrogen, Burlington, ON) based RNA extraction protocol was used. Briefly, 250 µL of whole blood was mixed with 50 µL of 5% saponin (Sigma-Aldrich, Oakville, ON) solution and kept at room temperature for 15 min. In this step, blood was mixed with saponin by shaking the tubes by hand, no vigorous mixing such as vortex mixing was applied. Then, saponin lysate was mixed and homogenized with 1500 µL of Trizol reagent (pH was adjusted to 7.2) and kept at room temperature for 10 min. Afterward, blood lysate was centrifuged at 4 °C and 10,000 rpm for 5 min and the supernatant decanted for downstream purification. The subsequent steps were performed according to the manufacturer’s instructions except for the final washing step (no 70% ethanol required) [31]. Two µL of the extract was used for each US-LAMP reaction. A detailed description of the protocol can be found in Additional file 3.

### Nucleic acid extraction–dried blood spots (DBS)

A modified total nucleic acid extraction protocol from DBS was used [24]. Fifty microliter of the whole blood sample was spotted onto Whatman 903 protein saver cards (GE Healthcare, Mississauga, ON) and allowed to air-dry overnight. A standard 6 mm-diameter hole puncher was used to cut spots into individual tubes. Dried blood containing filter paper pieces were mixed with lysis buffer [24] and incubated at 65 °C and 250 rpm shaking speed for 2.5 h in an orbital shaker. The 700 µL supernatant was transferred into HiBind^®^ DNA mini columns (Omega Bio-tek, Norcross, GA) and centrifuged at 2000 rpm for 2 min followed by another spinning step of 8000 rpm for 1 min. Column-bound nucleic acid was washed with 500 µL of the “wash buffer 1” by centrifuging the column at 8000 rpm for 1 min. Columns were washed with 500 µL of the “wash buffer 2” through centrifuging at 13,000 rpm for 3 min. An eluate of total nucleic acid with 50 µL of TE buffer (5 min wait after the addition of TE buffer) was obtained by centrifuging at 8000 rpm for 1 min. Ten microliters of the filter paper extract was used in a single US-LAMP reaction. A detailed description of the protocol can be found in Additional file 4.

### Ultrasensitive loop mediated amplification (US-LAMP) assay conditions

*Bst* 2.0 WarmStart^®^ DNA polymerase was combined with WarmStart^®^ reverse transcriptase in 1X Isothermal Amplification Buffer (New England Biolabs, Whitby, ON) to perform the US-LAMP assay. In a 25-µL LAMP reaction mixture, 1.6 µM F1P and B1P, 0.8 µM LPF and LPB, 0.2 µM F3 and B3 primer concentrations, 8 mM MgSO_4_, 1.4 mM dNTPs, 0.8 M Betaine (Sigma-Aldrich, Oakville, ON, Canada), 8 unit of *Bst* 2.0 WarmStart^®^ DNA Polymerase and 7.5 unit of Warm Start^®^ reverse transcriptase were used. The assay was optimized with pre-addition of 0.5 µL of 50X SYBR green (Invitrogen, Burlington, ON) in the reaction mixture. Amplification was measured based on increased relative fluorescence units (RFU) per minute in the CFX-96 Real-Time PCR detection system (Bio-Rad, Mississauga, ON). A threshold RFU value of 200 was chosen based on the background fluorescence levels. Optimization studies were performed to arrive at the ideal incubation of 63 °C for both Pan and Pf-LAMP assays. The final assay duration was fixed at 30 min for the Pan-LAMP assay and 60 min for the Pf-LAMP assay after optimizing the amplification curves using the CFX96 Real Time System. For visual detection of LAMP amplification, 1 µL of 0.35% (v/v) Gel green (Biotium, Freemont, CA), and 3 mM Hydroxynapthol blue (Sigma-aldrich, Oakville, ON), was added to the master mix [32]. Nuclease free water (VWR, Mississauga, ON) was used to constitute the final reaction volume. After amplification, the reaction tubes were exposed to blue LED light using a Blue Light Transilluminator (New England Biogroup, Atkinson, NH) to visualize the fluorescence due to the inter-chelation of gel green with the amplicons.

### Limit of detection (LOD) analysis

Uninfected blood, collected from a healthy donor, was spiked with in vitro culture of *P. falciparum* strain 3D7. A serial dilution of spiked blood with uninfected blood was made resulting in a parasite count range of 1 to 10,000 parasite/mL. Here, the parasite count was established by microscopy. Total nucleic acid was extracted from whole blood as well as from the DBS (50 µL) prepared from each dilution. Subsequently, the nucleic acid was amplified from the extracts by *Plasmodium* genus (Pan) and *P. falciparum* (Pf)-specific primers sets in the CFX-96 Real Time system. To detect real-time amplification, fluorescence measurement was taken every minute.

After initial assessment on culture-spiked blood, whole blood from one of each *P. falciparum, Plasmodium vivax* and *Plasmodium ovale* spp.-infected patients was diluted with healthy donor blood to obtain a parasite count ranging from 1 to 10,000 parasite/mL. Here, *P. falciparum* dilutions were tested in triplicate by both Pan and Pf-specific primers set while *P. vivax* and *P. ovale* spp. dilutions were tested in triplicate with only genus-specific primers. LODs were determined using the CFX-96 Real Time System subsequently confirmed through observing gel green fluorescence. If at least two out of three replicates of a certain dilution were tested positive by LAMP assays, the result was noted as positive.

### Stability studies

The stability of total nucleic acid, including RNA, in the DBS was evaluated. Multiple DBS were made from the serially diluted *P. falciparum* (3D7) culture spiked whole blood specimens. Total nucleic acid was extracted in duplicate from those spots at 3, 10, 17, 24, 30, 60, 90, 120 and 150 days and tested by genus-specific primers.

### Assay validation using clinical samples

A combination of whole blood specimens (symptomatic returning travellers to Calgary, Canada) and DBS (asymptomatic individuals from Ethiopia and Bangladesh) were used for validation of the assay. For symptomatic travellers, 41 malaria positive specimens and 72 negative specimens based on microscopy were obtained between September 2017 to May 2018 were used. Of the 41 positives, 24 were positive for *P. falciparum*, 12 were *P. vivax* positive and five were positive for *P. ovale* spp. Total nucleic acid was extracted from the fresh blood samples within 24 h of collection by the modified Trizol extraction mentioned above. Additionally, to validate the utility of US-LAMP for detection of low-level infections, DBS samples collected previously from asymptomatic individuals were obtained from a relatively high transmission area (Gondar, Ethiopia, n = 308) and a low transmission area (Bandarban, Bangladesh, n = 186). Whole blood samples were collected in EDTA tubes through venipuncture, and subsequently, 50 µL of the EDTA mixed blood specimens were spotted onto Whatman 903 protein saver card immediately after collection. Then, the blood spots were air-dried and stored at the room temperature in Calgary which is around 20–25 °C. Additionally, thick and thin blood smears were prepared for microscopy prior to the mixing with EDTA. Total nucleic acid was extracted from the DBS by the aforementioned protocol, and LAMP assays were conducted on the extracts with the pre-addition of gel green and hydroxynaphthol blue in the reaction mixture. Ethical approval were obtained from the corresponding Ethical Review board of University of Gondar (CMHS08/28/2013), International Center for Diarrheal Disease Research, Bangladesh (icddr,b:PR-15021), and University of Calgary Conjoint Health Research Ethics Board (REB17-2220). For sensitivity and specificity analysis, qRT-PCR [24] targeting 18S rRNA was used as the gold standard.

## Results

### Limit of detection (LOD)—culture spiked blood

A standard curve was obtained by plotting time to amplification against the logarithms of initial parasite count per mL. The goodness of fit to the straight line (R^2^) values were 0.813 and 0.641 respectively from whole blood and DBS. The data confirm that the *Plasmodium* genus-level US-LAMP assay consistently detects the presence of *P. falciparum* as low as 10 parasite/mL of culture-spiked whole blood, whereas the detection limit was 25 parasite/mL from DBS (Additional file 1: Figure S1).

### Limit of detection (LOD)—clinical specimens

**Fig. 1:**
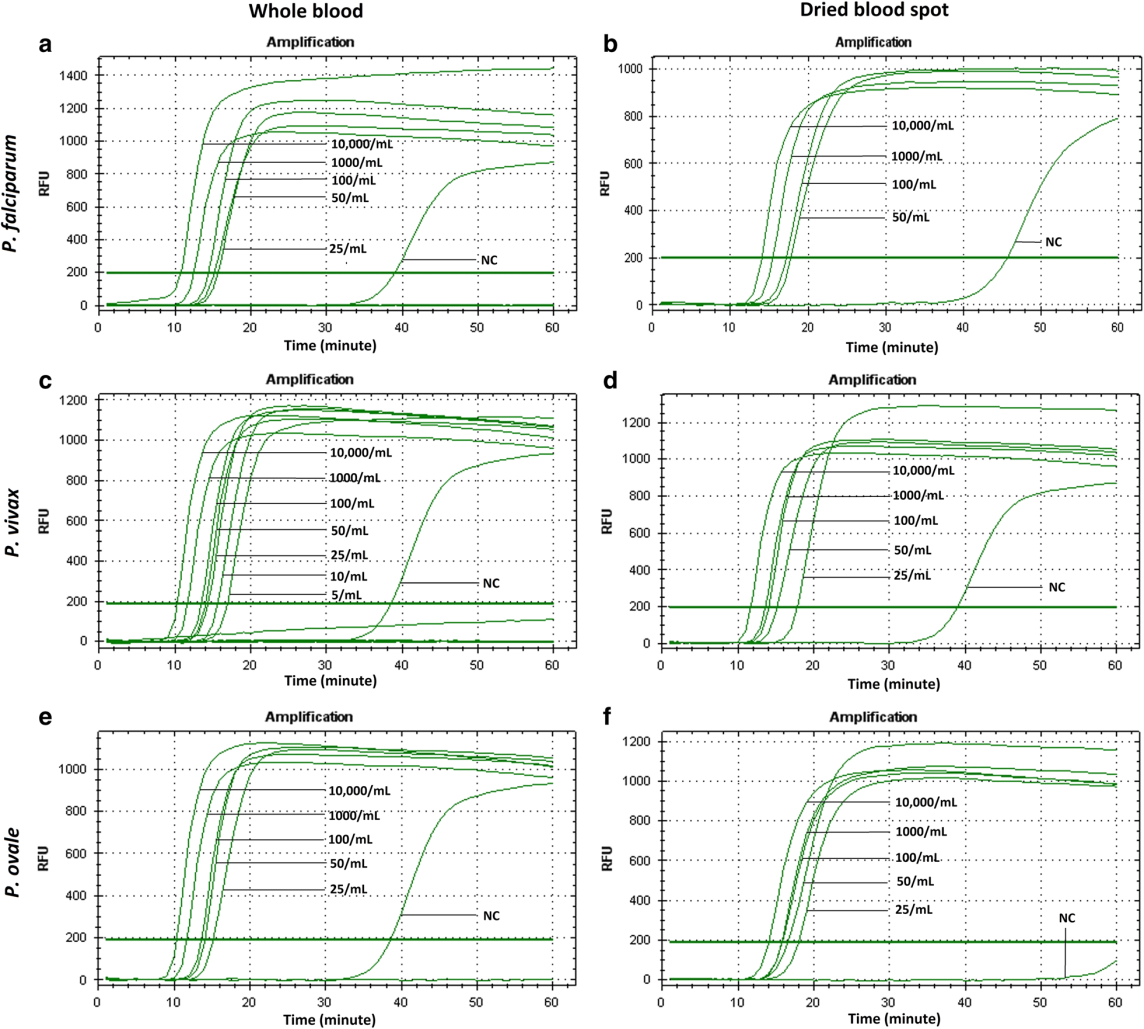
Amplification curve obtained from CFX96 Real Time system for Pan-LAMP assay. Here, **a**, **b** shows the amplification curve of *Plasmodium falciparum* dilution extracted from whole blood and DBS respectively (one representative experiment). Similarly, **c**–**f** describe the amplicons obtained for *Plasmodium vivax* and *Plasmodium ovale* spp. serial dilutions from whole blood and DBS extracts respectively. The threshold bar was fixed at 200 relative fluorescence units (RFU) to keep all background fluorescence levels under the bar. The numbers indicated next to each curve are parasite count/mL

**Fig. 2:**
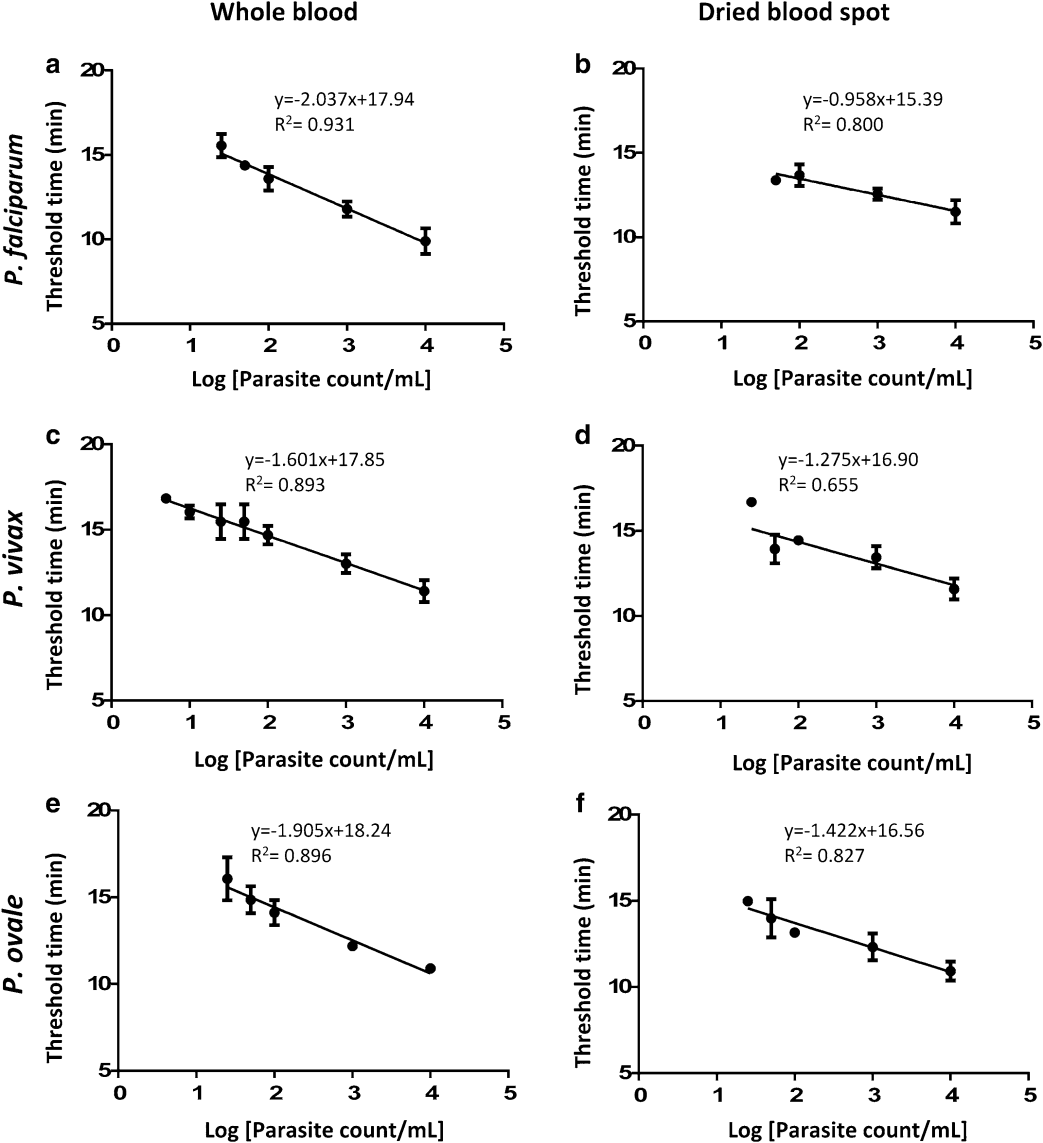
Validation of Pan US-LAMP assay on serially diluted clinical specimens. US-LAMP assay was carried on whole blood (**a**, **c**, **e**) and dried blood spot (DBS) extracted total nucleic acid (**b**, **d**, **f**). Here, **a**–**f** summarizes the data obtained from *P. falciparum*, *P. vivax* and *P. ovale* spp. serial dilutions prepared from clinical specimens. Data was obtained from triplicate experiments where error bars indicate standard error of mean (SEM). Threshold time (min = minutes) was determined by placing the threshold bar at 200 RFU in the CFX96 Real Time system. R^2^ indicates goodness of fit to the straight line

**Fig. 3:**
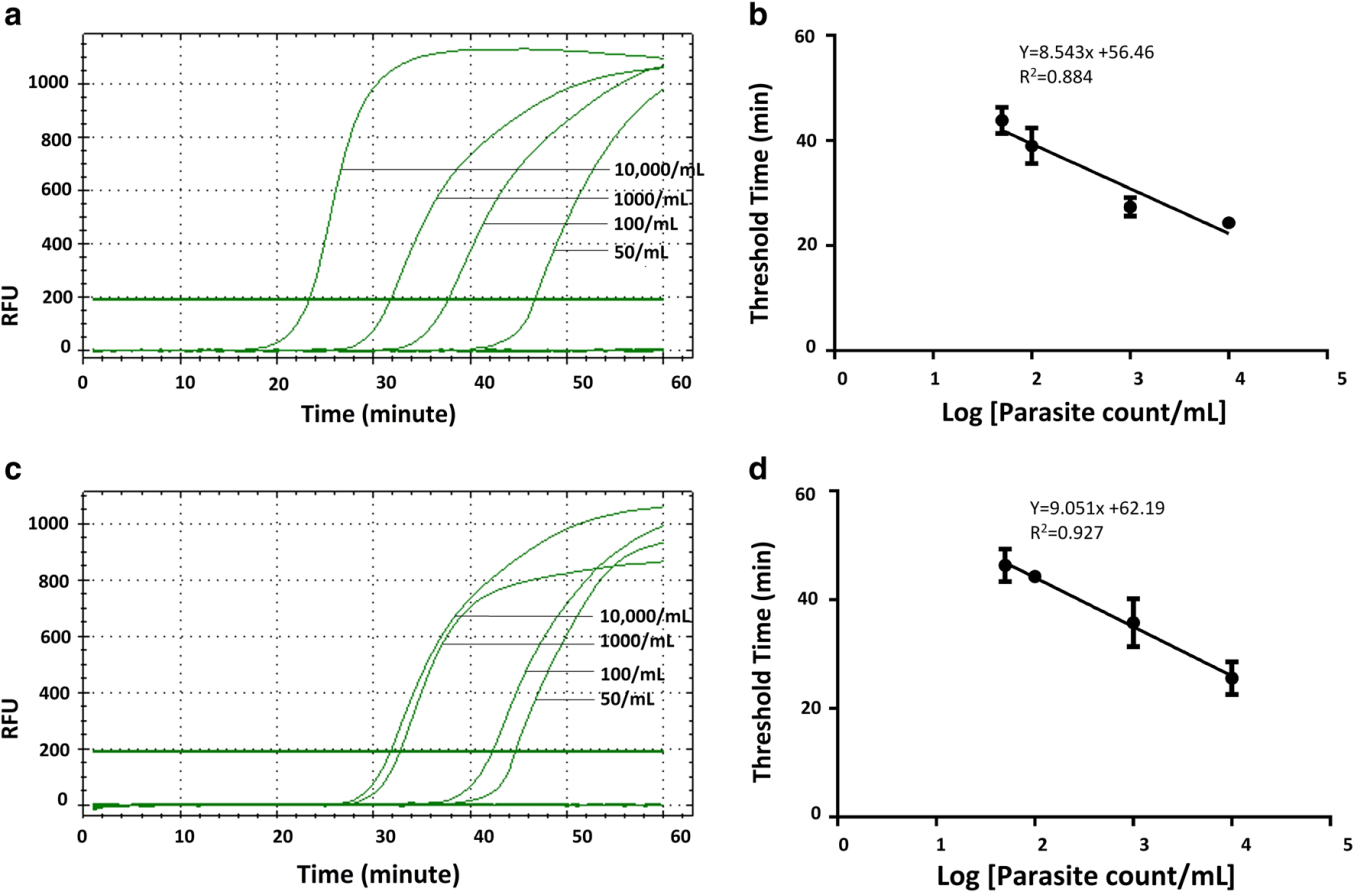
Validation of *P. falciparum* specific US-LAMP assay on diluted clinical specimens. Pf-LAMP was carried out on whole blood (**a**, **b**) and dried blood spot (**c**, **d**) extracted total nucleic acid. Experiments were performed in triplicate where error bars indicate standard error of mean (SEM). Here again, the threshold time (min = minutes) was determined by placing the threshold bar at 200 RFU in the CFX96 Real Time system. Each number/mL is indicative of parasite count/mL and R^2^ indicates goodness of fit to the straight line. **a** and **c** represents one of the three corresponding experiments

**Fig. 4:**
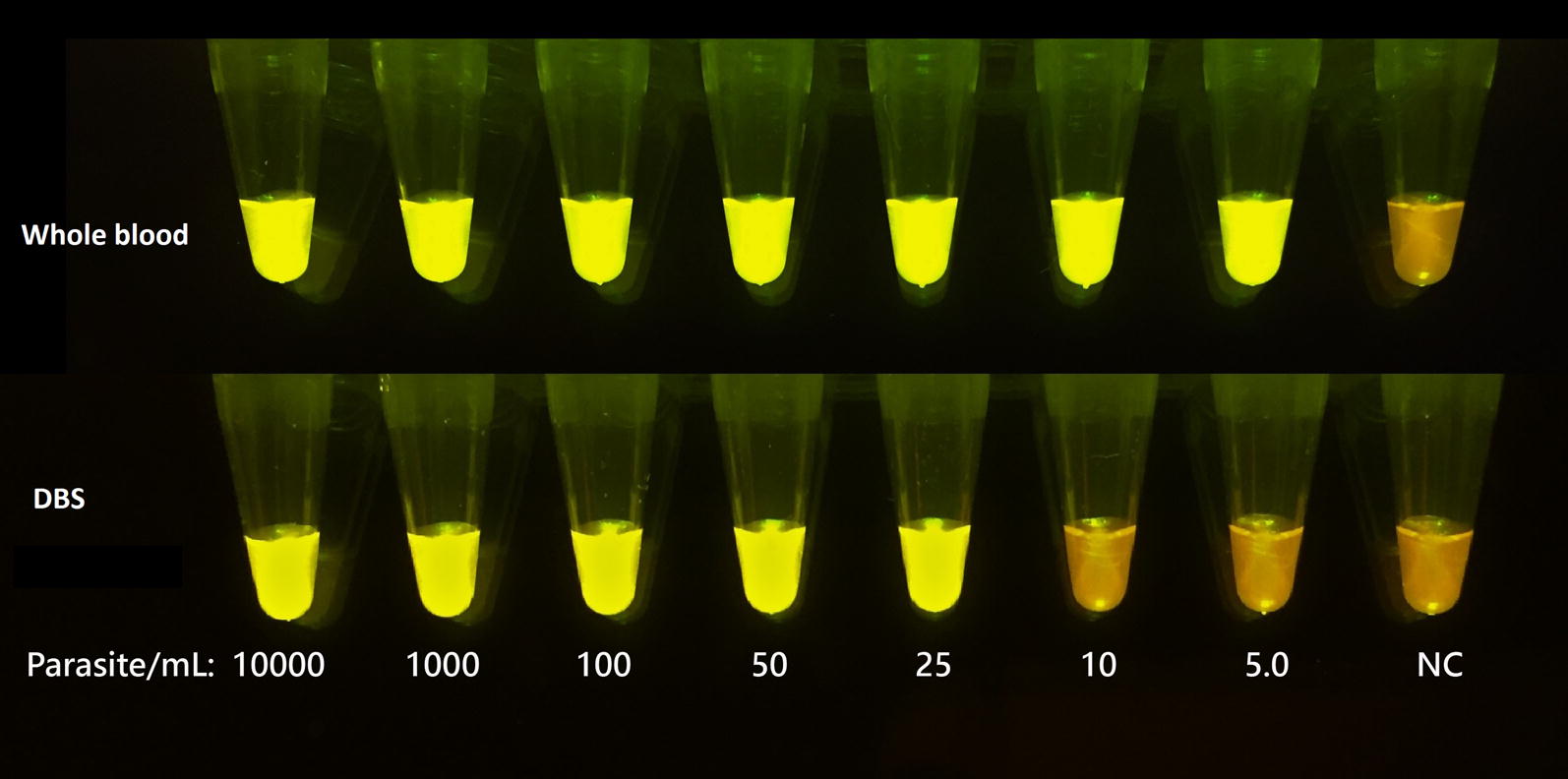
Observation of fluorescence by pre-addition of gel green in the reaction mixture. Here, results were shown for *P. vivax* dilutions. A representative experiment is shown. DBS stands for dried blood spot

Standard curves were plotted using threshold time against the logarithms of initial parasite count per mL for *P. falciparum* using genus- and species-specific primers to determine the assay dynamic range (Figs. 1 and 2). The LOD of 50 parasite/mL and 50–100 parasite/mL from whole blood and DBS in case of the *P. falciparum*-specific LAMP assay was achieved, with a goodness of fit to the straight line of 0.884 and 0.927, respectively (Fig. 3). Standard curves were plotted separately for whole blood and DBS using time to amplification (threshold time) against the logarithms of initial parasite count per mL for *P. falciparum*, *P. vivax*, and *P. ovale* spp. serial dilutions prepared from patient specimens. In the case of *P. falciparum,* LODs of 25 parasite/mL and 50–100 parasite/mL from whole blood and DBS were obtained, respectively (Fig. 1). Corresponding goodness of fit to the straight line values were 0.931 (whole blood) and 0.800 (DBS). LODs of 5–10 parasites/mL and 25–50 parasite/mL for *P. vivax* were obtained using whole blood and DBS (Fig. 2). LODs for *P. ovale* spp. were 25 parasites/mL from whole blood and 25–50 parasite/mL from DBS. The goodness of fit to the straight line was 0.896 and 0.827 for corresponding whole blood and DBS extracts of *P. ovale.* Through pre-addition of gel green, visual detection of amplification was also possible (Fig. 4). Additionally, data demonstrate that a maximum of four spots can be batched together without compromising the limit of detection (Additional file 1: Figure S2).

### Stability of the genetic material

From *P. falciparum* strain 3D7 culture-spiked blood, DNA and RNA are stable for at least 5 months on Whatman 903 protein saver card. However, best results were obtained within 1 month. After 1 month, inconsistent amplification from lower dilutions at 25 and 50 parasites/mL occurred (Table 2).

**Table 2:** Stability assessment of the nucleic acid on Whatmann 903 protein saver card (dried blood spots [DBS])

Parasite/mL	Day 03	Day 10	Day 17	Day 24	Day 31	Day 60	Day 90	Day 120	Day 150
10,000	++	++	++	++	++	++	++	++	++
1000	++	++	++	++	++	++	++	++	++
100	++	++	++	++	++	++	++	++	++
50	++	++	++	++	++	++	+−	++	+−
25	++	++	++	++	++	+−	++	++	+−
10	−−	−−	−−	−−	−−	−−	−−	−−	−−

Samples were extracted and tested in duplicate, each positive and negative symbol is indicative of the result from each Pan-LAMP assay on *P. falciparum*

### Validation of US-LAMP

An initial verification study was performed on a set of clinical specimens from symptomatic returning travellers. Pan-LAMP and Pf-LAMP assays were 100% (95% CI 82.8–100) sensitive for the detection of symptomatic malaria, whereas specificity was 98.6% (95% CI 91.5–99.9) and 97.8% (95% CI 91.4–99.6) for pan-LAMP and Pf-LAMP assay, respectively (Table 3). Subsequently, DBS samples from asymptomatic individuals in Gondar (Ethiopia) and Bandarban (Bangladesh) were used to validate the assay on low-level infections. Overall, Pan-LAMP was 97% sensitive (95% CI 82.5–99.8) and 99.1% (95% CI 97.6–99.7) specific for identifying asymptomatic *Plasmodium* infection while Pf-LAMP was concluded to be 100% sensitive and 99.8% specific for asymptomatic *P. falciparum* cases (Table 3). QRT-PCR and Pan-LAMP detected 29 and 32 positives, respectively, from the 308 microscopy negative DBS samples obtained from Gondar. The additional infections detected by US-LAMP but not microscopy comprised 10 *P. falciparum,* 16 *P. vivax*, and 3 *P. falciparum* and *P. vivax* mixed infections. US-LAMP detected one additional *P. falciparum* asymptomatic carrier not identified by microscopy from the Bandarban region (Additional file 2: Table S1). Detailed sensitivity and specificity calculations can be found in the Additional file 2: Tables S2–S5.

**Table 3:** Sensitivity and specificity of the US-LAMP assay on symptomatic and asymptomatic specimen compared to RT-PCR

Sample type	Assay type	Sensitivity (%)	95% confidence interval (CI)	Specificity (%)	95% confidence interval (CI)	Positive predictive value (PPV) (%)	95% confidence interval (CI)	Negative predictive value (NPV) (%)	95% confidence interval (CI)
Symptomatic Calgary (N = 113)	Pan-LAMP	100	89.3–100	98.6	91.5–99.9	97.6	85.9–99.9	100	93.6–100
	Pf-LAMP	100	82.8–100	97.8	91.4–99.6	92.3	73.4–98.7	100	94.7–100
Asymptomatic Bangladesh (N = 186)	Pan-LAMP	100	39.6–100	100	97.4–100	100	39.6–100	100	97.4–100
	Pf-LAMP	100	39.6–100	100	97.4–100	100	39.6–100	100	97.4–100
Asymptomatic Ethiopia (N = 308)	Pan-LAMP	96.6	80.4–99.8	98.6	96.1–99.5	87.5	70.0–95.9	99.6	97.7–100
	Pf-LAMP	100	69.9–100	99.7	97.8–100	92.3	62.1–99.6	100	98.4–100
Overall asymptomatic (N = 494)	Pan-LAMP	97	82.5–99.8	99.1	97.6–99.7	88.9	73.0–96.4	99.7	98.6–100
	Pf-LAMP	100	75.9–100	99.8	98.6–100	94.1	69.2–99.7	100	99.0–100

## Discussion

Many LMIC do not have the laboratory infrastructure, training, or access to reagents to perform ultrasensitive PCR methods. LAMP with its minimal requirement of a heat block and visual read out provides a useful alternative for active surveillance of malaria in a population where elimination is being considered. This work describes the first easy-to-perform, low cost ultrasensitive LAMP assay (LOD below 100 parasites per mL) for malaria detection from DBS. Samples can be collected in the form of DBS from remote endemic areas, transported to a regional laboratory, preserved at room temperature for several months, and then tested. The assay achieved 100% sensitivity in detecting symptomatic malaria cases while maintaining a very high level of specificity (> 97%). More importantly, US-LAMP demonstrated excellent sensitivity (> 97%) and specificity (> 99%) for detecting very low-level asymptomatic infections present in both high and low transmission settings in Africa and Asia. Here, an additional 32 out of 308 asymptomatic malaria infections (10.4%) were detected by US-LAMP in Gondar (Ethiopia) where moderate to high transmission malaria occurs highlighting the value ultrasensitive detection for elimination, as reviewed recently by Lindblade et al. [9]. In earlier studies, US-LAMP amplified *exp1* mRNA concentrated from a large volume (2 mL) of fresh whole blood [25]. However, this is practically difficult to obtain at the field level especially from younger children. This assay is only applicable for detecting *P. falciparum* cases not for identifying other species. Moreover, the stability of the mRNA transcript from the *exp1* gene was not studied.

In low transmission settings, to save reagents, DBS samples can be batched into groups of four for initial screening and subsequently only positive batches selected for individual testing. The batch approach is particularly useful for the detection of asymptomatic malaria in large-scale surveys where the positivity rate is expected to be low. Additionally, samples testing positive can be further assessed for gametocyte carriage by a *Pfs25*-specific LAMP assay if required [33].

Primers targeting *P. falciparum* were modified specifically to amplify 18S rRNA located in chromosome 5 and 7. The latter loci are known to be highly expressed in the blood stages of the *P. falciparum* [23]. By using total nucleic acid (18S RNA and DNA) as the target for amplification instead of DNA only, a LOD of 5–50 parasite/mL in whole blood could be attained [13]. This is a 1000- to 10,000-fold improvement in LOD compared to previous LAMP assays targeting 18S rDNA alone. Data exhibited that genus-level ‘Pan” primers had the best LOD in the case of *P. vivax* (5–10 parasites/mL) and *P. falciparum* (10 parasites/mL) using whole blood. LOD analysis is based on a culture-derived sample which may not reflect actual patient samples. This study evaluated the Pan-LAMP assay for three species: *P. falciparum*, *P. vivax* and *P. ovale*. A limitation is that the assay on *Plasmodium malariae* and *Plasmodium knowlesi* could not be tested due to lack of sample availability. Qualitative detection of fluorescence created by the gel green was robust and closely mirrored results using a fluorescence detection system. Visual detection again makes the assay more amenable to a resource-limited setting. Another limitation is the reliance on a labour-intensive column-based RNA extraction protocol which prevents the use of this assay at a health centre but is more suitable for a regional laboratory. An additional dimension could be added in the study by comparing the US-LAMP assay with a DNA-based commercial LAMP assay. However, this strategy was omitted due to the shortage of sample volume. However, the LODs achieved through the US-LAMP assay are better than the commercially available DNA-based LAMP assays.

## Conclusion

In summary, the US-LAMP assay presented here is robust, cost-effective, and relatively simple for surveillance of asymptomatic malaria cases that are low-level and comprise the infectious reservoir. Further improvements are required to simplify the nucleic acid extraction process, ideally on a microfluidic cartridge.

## Supplementary information

**Additional file 1: Figure S1.** Initial optimization of the genus-specific (pan) US-LAMP assay on 3D7 culture spiked whole blood. **Figure S2.** Gel green fluorescence observed after Pan-LAMP assay on *Plasmodium ovale* spp. dilutions from batched total nucleic acid extraction approach.

**Additional file 2: Table S1.** Details of the asymptomatic samples tested positive by RT-qPCR, Pan-LAMP and Pf-LAMP. **Table S2.** 2 × 2 table for sensitivity and specificity calculation from symptomatic samples (returning travellers in Calgary). **Table S3.** 2 × 2 table for sensitivity and specificity calculation from asymptomatic (Bandarban) samples. **Table S4.** 2 × 2 table for sensitivity and specificity calculation from asymptomatic (Gondar) samples. **Table S5.** 2 × 2 table for sensitivity and specificity calculation from all asymptomatic (Gondar + Bandarban)) samples.

**Additional file 3.** Total nucleic acid extraction from filter paper dried blood spots.

**Additional file 4.** Reverse transcriptase Real Time PCR protocol for dried blood spot extracted samples.

## Data Availability

The datasets generated during and/or analysed during the current study are available from the corresponding author on reasonable request.

## Abbreviations

DBS: dried blood spot
EDTA: ethylene diamine tetra-acetic acid
LOD: limit of detection
LAMP: loop-mediated Isothermal amplification
PCR: polymerase chain reaction
Pf: *P. falciparum*
qPCR: quantitative real time PCR
qRT-PCR: quantitative real time reverse transcriptase PCR
RDT: rapid diagnostic test
TE buffer: tris–EDTA buffer
rRNA: ribosomal RNA
US-LAMP: ultrasensitive LAMP

## References

[1] World Health Organization (2017). World malaria report 2017.

[2] Poirot E, Skarbinski J, Sinclair D, Kachur SP, Slutsker L, Hwang J (2013). Mass drug administration for malaria. Cochrane Database Syst Rev..

[3] McMorrow M, Aidoo M, Kachur S (2011). Malaria rapid diagnostic tests in elimination settings—can they find the last parasite?. Clin Microbiol Infect.

[4] Milne L, Kyi M, Chiodini P, Warhurst D (1994). Accuracy of routine laboratory diagnosis of malaria in the United Kingdom. J Clin Pathol.

[5] Wongsrichanalai C, Barcus MJ, Muth S, Sutamihardja A, Wernsdorfer WH (2007). A review of malaria diagnostic tools: microscopy and rapid diagnostic test (RDT). Am J Trop Med Hyg.

[6] Landier J, Haohankhunnatham W, Das S, Konghahong K, Christensen P, Raksuansak J (2018). Operational performance of a *Plasmodium falciparum* ultrasensitive rapid diagnostic test for the detection of asymptomatic infections in Eastern Myanmar. J Clin Microbiol.

[7] Imwong M, Stepniewska K, Tripura R, Peto TJ, Lwin KM, Vihokhern B (2015). Numerical distributions of parasite densities during asymptomatic malaria. J Infect Dis.

[8] Chen I, Clarke SE, Gosling R, Hamainza B, Killeen G, Magill A (2016). “Asymptomatic” malaria: a chronic and debilitating infection that should be treated. PLoS Med..

[9] Lindblade KA, Steinhardt L, Samuels A, Kachur SP, Slutsker L (2013). The silent threat: asymptomatic parasitemia and malaria transmission. Expert Rev Anti Infect Ther..

[10] Hopkins H, González IJ, Polley SD, Angutoko P, Ategeka J, Asiimwe C (2013). Highly sensitive detection of malaria parasitemia in a malaria-endemic setting: performance of a new loop-mediated isothermal amplification kit in a remote clinic in Uganda. J Infect Dis.

[11] Polley SD, González IJ, Mohamed D, Daly R, Bowers K, Watson J (2013). Clinical evaluation of a loop-mediated amplification kit for diagnosis of imported malaria. J Infect Dis.

[12] Mohon AN, Lee LDY, Bayih AG, Folefoc A, Guelig D, Burton RA (2016). NINA-LAMP compared to microscopy, RDT, and nested PCR for the detection of imported malaria. Diagn Microbiol Infect Dis..

[13] Han E-T, Watanabe R, Sattabongkot J, Khuntirat B, Sirichaisinthop J, Iriko H (2007). Detection of four *Plasmodium* species by genus-and species-specific loop-mediated isothermal amplification for clinical diagnosis. J Clin Microbiol.

[14] Lucchi NW, Demas A, Narayanan J, Sumari D, Kabanywanyi A, Kachur SP (2010). Real-time fluorescence loop mediated isothermal amplification for the diagnosis of malaria. PLoS ONE.

[15] Mohon AN, Menard D, Alam MS, Perera K, Pillai DR (2018). A novel single-nucleotide polymorphism loop mediated isothermal amplification assay for detection of artemisinin-resistant *Plasmodium falciparum* malaria. Open Forum Infect Dis..

[16] Chahar M, Mishra N, Anvikar A, Dixit R, Valecha N (2017). Establishment and application of a novel isothermal amplification assay for rapid detection of chloroquine resistance (K76T) in *Plasmodium falciparum*. Sci Rep..

[17] Lucchi NW, Gaye M, Diallo MA, Goldman IF, Ljolje D, Deme AB (2016). Evaluation of the illumigene malaria LAMP: a robust molecular diagnostic tool for malaria parasites. Sci Rep..

[18] Rypien C, Chow B, Chan W, Church D, Pillai DR (2017). Detection of *Plasmodium* spp. infection by the illumigene malaria assay compared to reference microscopy and real-time PCR. J Clin Microbiol..

[19] Perera RS, Ding XC, Tully F, Oliver J, Bright N, Bell D (2017). Development and clinical performance of high throughput loop-mediated isothermal amplification for detection of malaria. PLoS ONE.

[20] Aydin-Schmidt B, Morris U, Ding XC, Jovel I, Msellem MI, Bergman D (2017). Field evaluation of a high throughput loop mediated isothermal amplification test for the detection of asymptomatic *Plasmodium* infections in Zanzibar. PLoS ONE.

[21] Snounou G, Viriyakosol S, Zhu XP, Jarra W, Pinheiro L, Rosario VED (1993). High sensitivity of detection of human malaria parasites by the use of nested polymerase chain reaction. Mol Biochem Parasitol..

[22] Imwong M, Hanchana S, Malleret B, Rénia L, Day NP, Dondorp A (2014). High throughput ultra-sensitive molecular techniques to quantify low density malaria parasitaemias. J Clin Microbiol.

[23] Murphy SC, Prentice JL, Williamson K, Wallis CK, Fang FC, Fried M (2012). Real-time quantitative reverse transcription PCR for monitoring of blood-stage *Plasmodium falciparum* infections in malaria human challenge trials. Am J Trop Med Hyg.

[24] Zainabadi K, Adams M, Han ZY, Lwin HW, Han KT, Ouattara A (2017). A novel method for extracting nucleic acids from dried blood spots for ultrasensitive detection of low-density *Plasmodium falciparum* and *Plasmodium vivax* infections. Malar J..

[25] Kemleu S, Guelig D, Moukoko CE, Essangui E, Diesburg S, Mouliom A (2016). A field-tailored reverse transcription loop-mediated isothermal assay for high sensitivity detection of *Plasmodium falciparum* infections. PLoS ONE.

[26] Waters A, McCutchan T (1989). Partial sequence of the asexually expressed SU rRNA gene of *Plasmodium vivax*. Nucleic Acids Res.

[27] Li J, Gutell RR, Damberger SH, Wirtz RA, Kissinger JC, Rogers MJ (1997). Regulation and trafficking of three distinct 18 S ribosomal RNAs during development of the malaria parasite. J Mol Biol.

[28] Chakrabarti K, Pearson M, Grate L, Sterne-Weiler T, Deans J, Donohue JP (2007). Structural RNAs of known and unknown function identified in malaria parasites by comparative genomics and RNA analysis. RNA.

[29] Mohon AN, Elahi R, Khan WA, Haque R, Sullivan DJ, Alam MS (2014). A new visually improved and sensitive loop mediated isothermal amplification (LAMP) for diagnosis of symptomatic falciparum malaria. Acta Trop.

[30] McCutchan T, Li J, McConkey G, Rogers M, Waters A (1995). The cytoplasmic ribosomal RNAs of *Plasmodium* spp. Parasitol Today..

[31] Trizol Reagent-User Guide. https://tools.thermofisher.com/content/sfs/manuals/trizol_reagent.pdf. Accessed 7 Oct 2019.

[32] Hayashida K, Kajino K, Hachaambwa L, Namangala B, Sugimoto C (2015). Direct blood dry LAMP: a rapid, stable, and easy diagnostic tool for Human African Trypanosomiasis. PLoS Negl Trop Dis..

[33] Buates S, Bantuchai S, Sattabongkot J, Han E-T, Tsuboi T, Udomsangpetch R (2010). Development of a reverse transcription-loop-mediated isothermal amplification (RT-LAMP) for clinical detection of *Plasmodium falciparum* gametocytes. Parasitol Int.

