# Supplementary material for: Ultrasensitive loop mediated isothermal amplification (US-LAMP) to detect malaria for elimination
Source: Malar J. 2019 Oct 16;18:350. doi: 10.1186/s12936-019-2979-4 (PMC6796404; doi:10.1186/s12936-019-2979-4)
Supplement: Supplementary file 1 — Additional file 1: Figure S1. Initial optimization of the genus-specific (pan) US-LAMP assay on 3D7 culture spiked whole blood. Figure S2. Gel green fluorescence observed after Pan-LAMP assay on Plasmodium ovale spp. dilutions from batched total nucleic acid extraction approach. [file 12936_2019_2979_MOESM1_ESM.docx]

**Additional Figures:**


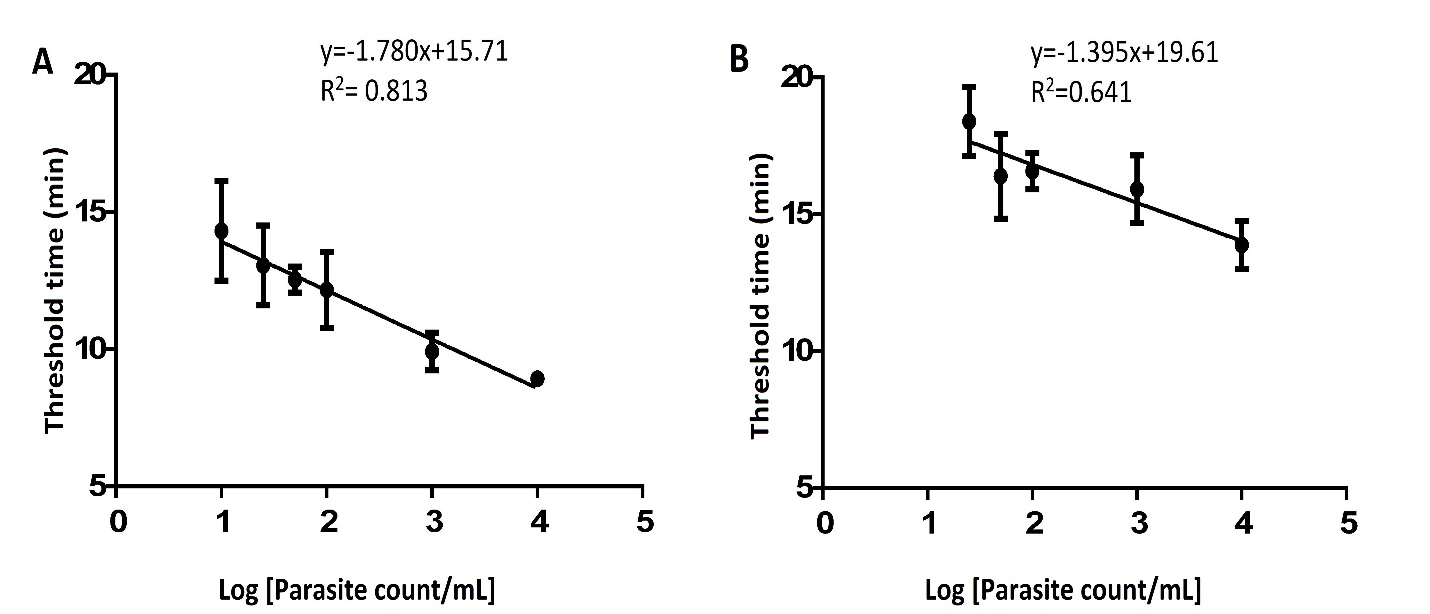


*R^2^ indicates the goodness of fit to the straight line

Figure S1: Initial optimization of the genus-specific (pan) US-LAMP assay on 3D7 culture spiked whole blood. Here, reaction time was plotted against log parasite count per mL. Results were obtained through US-LAMP assay using Trizol-Saponin extracted total nucleic acid from whole blood (A) and spin-column extracted total nucleic acid from filter paper dried blood spots (B). Experiments were performed in triplicate and error bars in the figure indicate standard error of mean (SEM). Threshold time (min=minutes) was determined by placing the threshold bar at 200 relative fluorescence units (RFU) in the CFX96 Real Time system.


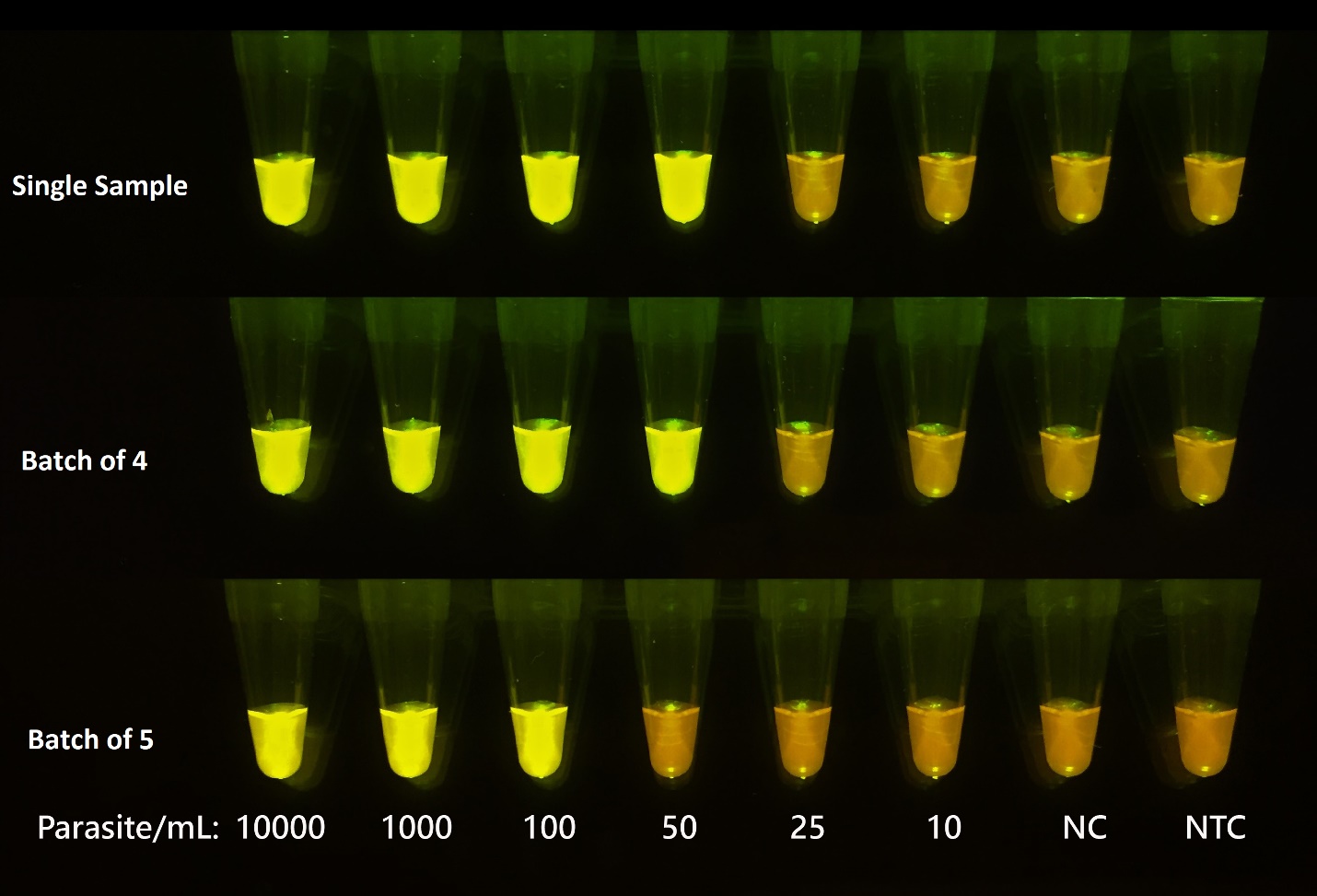


Figure S2: Gel green fluorescence observed after Pan-LAMP assay on *P. ovale* spp. dilutions from batched total nucleic acid extraction approach. Here, top row shows the results from a typical single spot assay (LOD; 25-50 parasites/mL); middle row depicts the results obtained after combination of three negative control spots with one positive spot of variable parasite count. Similarly, the bottom row delineates the outcome of the combination of one positive spot with four negative control spots. NTC stands for no template control (water control). This figure is one representative of a triplicate experiment.
