# Supplementary material for: Ultrasensitive loop mediated isothermal amplification (US-LAMP) to detect malaria for elimination
Source: Malar J. 2019 Oct 16;18:350. doi: 10.1186/s12936-019-2979-4 (PMC6796404; doi:10.1186/s12936-019-2979-4)
Supplement: Supplementary file 2 — Additional file 2: Table S1. Details of the asymptomatic samples tested positive by RT-qPCR, Pan-LAMP and Pf-LAMP. Table S2. 2 × 2 table for sensitivity and specificity calculation from symptomatic samples (returning travellers in Calgary). Table S3. 2 × 2 table for sensitivity and specificity calculation from asymptomatic (Bandarban) samples. Table S4. 2 × 2 table for sensitivity and specificity calculation from asymptomatic (Gondar) samples. Table S5. 2 × 2 table for sensitivity and specificity calculation from all asymptomatic (Gondar + Bandarban)) samples. [file 12936_2019_2979_MOESM2_ESM.docx]

Table S1: Details of the asymptomatic samples tested positive by RT-qPCR, Pan-LAMP and Pf-LAMP.

| Sample ID | Region | Pan-LAMP | Pf-LAMP | RT-PCR_Pf | RT-PCR_Pv | Microscopy | Pf  density/mL | Pv  density/mL |
| --- | --- | --- | --- | --- | --- | --- | --- | --- |
| MT144 | Gondar | Pos | Pos | Pos | Pos | Neg | 5555 | 31,622.80 |
| MK127 | Gondar | Pos | Pos | Pos | Pos | Neg | 135.8 | 649.2 |
| SN078 | Gondar | Pos | Pos | Pos | Pos | Neg | 12.7 | 263.1 |
| MT149 | Gondar | Pos | Pos | Pos | Neg | Neg | 49.1 | 0 |
| MK092 | Gondar | Pos | Pos | Pos | Neg | Neg | 127.3 | 0 |
| MK146 | Gondar | Pos | Pos | Pos | Neg | Neg | 16.8 | 0 |
| Mk183 | Gondar | Pos | Pos | Pos | Neg | Neg | 3392 | 0 |
| SN004 | Gondar | Pos | Pos | Pos | Neg | Neg | 92 | 0 |
| SN015 | Gondar | Pos | Pos | Pos | Neg | Neg | 336.2 | 0 |
| SN025 | Gondar | Pos | Pos | Pos | Neg | Neg | 66.2 | 0 |
| SN048 | Gondar | Pos | Pos | Pos | Neg | Neg | 116.3 | 0 |
| SN055 | Gondar | Pos | Pos | Pos | Neg | Neg | 28.4 | 0 |
| BD01-05 | Bandarban | Pos | Pos | Pos | Neg | Pos | 90.2 | 0 |
| BD03-18 | Bandarban | Pos | Pos | Pos | Neg | Pos | 1756 | 0 |
| BD20-35 | Bandarban | Pos | Pos | Pos | Neg | Pos | 11,040 | 0 |
| BD24-01 | Bandarban | Pos | Pos | Pos | Neg | Neg | 190.4 | 0 |
| SN058 | Gondar | Pos | Neg | Neg | Pos | Neg | 0 | 5011.9 |
| MK178 | Gondar | Neg | Pos | Neg | Neg | Neg | 0 | 0 |
| MT076 | Gondar | Pos | Neg | Neg | Pos | Neg | 0 | 251.2 |
| MT087 | Gondar | Pos | Neg | Neg | Pos | Neg | 0 | 199.5 |
| MT128 | Gondar | Pos | Neg | Neg | Pos | Neg | 0 | 158.6 |
| MT132 | Gondar | Pos | Neg | Neg | Pos | Neg | 0 | 501.8 |
| MT133 | Gondar | Pos | Neg | Neg | Pos | Neg | 0 | 125 |
| MT139 | Gondar | Pos | Neg | Neg | Pos | Neg | 0 | 15.84 |
| MT148 | Gondar | Pos | Neg | Neg | Pos | Neg | 0 | 134.9 |
| MT189 | Gondar | Pos | Neg | Neg | Pos | Neg | 0 | 794.3 |
| MK082 | Gondar | Pos | Neg | Neg | Pos | Neg | 0 | 749.9 |
| MK097 | Gondar | Pos | Neg | Neg | Pos | Neg | 0 | 631 |
| MK140 | Gondar | Pos | Neg | Neg | Pos | Neg | 0 | 223.9 |
| MK141 | Gondar | Pos | Neg | Neg | Pos | Neg | 0 | 166 |
| MK209 | Gondar | Pos | Neg | Neg | Pos | Neg | 0 | 116.5 |
| SN013 | Gondar | Pos | Neg | Neg | Pos | Neg | 0 | 47,863 |
| SN082 | Gondar | Pos | Neg | Neg | Pos | Neg | 0 | 103.2 |
| MK088 | Gondar | Neg | Neg | Neg | Pos | Neg | 0 | 58.1 |
| MK087 | Gondar | Pos | Neg | Neg | Neg | Neg | 0 | 0 |
| MK145 | Gondar | Pos | Neg | Neg | Neg | Neg | 0 | 0 |
| SN014 | Gondar | Pos | Neg | Neg | Neg | Neg | 0 | 0 |
| SN050 | Gondar | Pos | Neg | Neg | Neg | Neg | 0 | 0 |

*^Parasite density/mL data were retrieved by comparing the threshold cycles (Ct) values with the standard curve generated in RT-qPCR. Shaded samples IDs reflect internally discordant results by US-LAMP.^

Table S2: 2×2 table for sensitivity and specificity calculation from symptomatic samples (returning travelers in Calgary)

| Pan-LAMP vs RT-qPCR | | | | | Pf-LAMP vs RT-qPCR | | | | |
| --- | --- | --- | --- | --- | --- | --- | --- | --- | --- |
| Method | RT-PCR | | | | Method | RT-PCR | | | |
|  |  | Pos | Neg | Total |  |  | Pos | Neg | Total |
| Pan-LAMP | Pos | 41 | 1 | 42 | Pf-LAMP | Pos | 24 | 2 | 26 |
|  | Neg | 0 | 71 | 21 |  | Neg | 0 | 87 | 87 |
|  | Total | 41 | 72 | 113 |  | Total | 24 | 89 | 113 |

Table S3: 2×2 table for sensitivity and specificity calculation from asymptomatic (Bandarban) samples

| Pan-LAMP vs RT-qPCR | | | | | Pf-LAMP vs RT-qPCR | | | | |
| --- | --- | --- | --- | --- | --- | --- | --- | --- | --- |
| Method | RT-PCR | | | | Method | RT-PCR | | | |
|  |  | Pos | Neg | Total |  |  | Pos | Neg | Total |
| Pan-LAMP | Pos | 4 | 0 | 4 | Pf-LAMP | Pos | 4 | 0 | 4 |
|  | Neg | 0 | 182 | 182 |  | Neg | 0 | 182 | 182 |
|  | Total | 4 | 182 | 186 |  | Total | 4 | 182 | 186 |

Table S4: 2×2 table for sensitivity and specificity calculation from asymptomatic (Gondar) samples

| Pan-LAMP vs RT-qPCR | | | | | Pf-LAMP vs RT-qPCR | | | | |
| --- | --- | --- | --- | --- | --- | --- | --- | --- | --- |
| Method | RT-PCR | | | | Method | RT-PCR | | | |
|  |  | Pos | Neg | Total |  |  | Pos | Neg | Total |
| Pan-LAMP | Pos | 28 | 4 | 32 | Pf-LAMP | Pos | 12 | 1 | 13 |
|  | Neg | 1 | 275 | 276 |  | Neg | 0 | 295 | 295 |
|  | Total | 29 | 279 | 308 |  | Total | 12 | 296 | 308 |

Table S5: 2×2 table for sensitivity and specificity calculation from all asymptomatic (Gondar + Bandarban)) samples

| Pan-LAMP vs RT-qPCR | | | | | Pf-LAMP vs RT-qPCR | | | | |
| --- | --- | --- | --- | --- | --- | --- | --- | --- | --- |
| Method | RT-PCR | | | | Method | RT-PCR | | | |
|  |  | Pos | Neg | Total |  |  | Pos | Neg | Total |
| Pan-LAMP | Pos | 32 | 4 | 36 | Pf-LAMP | Pos | 16 | 1 | 17 |
|  | Neg | 1 | 457 | 458 |  | Neg | 0 | 477 | 477 |
|  | Total | 33 | 461 | 494 |  | Total | 16 | 478 | 494 |

*^For Table (S1-S5): Pos=Positive and Neg=Negative^
