# Supplementary material for: Ultrasensitive loop mediated isothermal amplification (US-LAMP) to detect malaria for elimination
Source: Malar J. 2019 Oct 16;18:350. doi: 10.1186/s12936-019-2979-4 (PMC6796404; doi:10.1186/s12936-019-2979-4)
Supplement: Supplementary file 3 — Additional file 3. Total nucleic acid extraction from filter paper dried blood spots. [file 12936_2019_2979_MOESM3_ESM.docx]

Hazards associated with equipment/machinery/materials/technique/process

- Guanidine thiocyanate, 2-mecrceptoethanol, Hydrochloric acid
- Hazardous in case of skin contact, of eye contact, of inhalation or ingestion.

Personal protective equipment

- Personnel handling chemical must wear proper personal protective equipment; laboratory coat, nitrile gloves and closed-toe shoes
- Fume hood

Emergency procedures

- Users must be familiar with the Emergency response procedures, Use of Emergency eye wash/ shower station, Spill Response Procedure and use of the Spill response kit.

**Composition of the lysis buffer:**

3M Guanidine thiocyanate

16.7% Isopropanol

2% Triton X100

10mM EDTA

5mM Tris-HCl pH 7.4

0.1% 6N HCl

0.5% 2-mercaptoethanol (pH 6.0-6.5)

**Composition of wash buffer-1: Same as lysis buffer except no 2-merceptoethanol**

**Composition of wash buffer-2:**

25% Ethanol

25% Isopropanol

100mM Sodium Chloride

10mM Trizma HCl pH 7.4

**Step by step procedures for task**

1. Diluting HCl from stock (20mL solution): (**Must be done inside a fume hood)**

- Usually, we have 36-38% HCl (Sigma-Aldrich) which equivalent to 11.65N concentration
- Take 9.65mL distilled water in a 50mL falcon tube
- Take 10.35 mL HCl from the stock by serological pipette
- Add dropwise to top of the water in the falcon tube
- Mix by shaking while adding HCl on water

1. Preparation of 1M Tris-HCl (pH7.4)

- Measure 12.14gm Trizma base for 100mL preparation and put in a beaker
- Add distilled water up to 95 mL
- Add the magnetic rod and mix the solution on the magnetic stirrer
- Check the pH and keep the pH electrode inside the solution
- Add 6N HCl dropwise and check the pH change
- When pH is stable at 7.4 stop adding HCl
- Transfer the solution in to a 100ML measuring cylinder
- Add water to make final volume up to 100mL and store in a bottle

1. Wash buffer-1 preparation

- Measure 70.9 gm Guanidine thiocyanate and put in in bottle (250 mL minimum capacity)
- Add 4.0mL of 0.5M EDTA
- Add 4.0mL TritonX100
- Add 1.0 mL of 1M Tris-HCl (pH7.4)
- Add 33.4 mL Isopropanol
- Add 200µL of 6N HCl **(do inside the fume hood)**
- Add 80.0mL distilled water
- Mix by a magnetic stirrer
- Transfer to a measuring cylinder and make the volume up to 200 mL
- Transfer to the bottle again and mix by inversion

1. Lysis buffer preparation

- Transfer 100mL of Wash buffer-1 in a new bottle
- Add 500µL 2-mercertoethanol **(this step must be done inside a fume hood)**
- Keep at room temperature
- Never open the Lysis buffer outside of fume hood

**Waste disposal procedures**

- liquid and solid waste must be disposed of according to the University of Calgary Hazardous Material Disposal Manual procedures through Chematix.

**Maintenance**

Clean the fume hood and centrifuge with 75% ethanol after completion with a paper towel and spray bottle.

**Step by step procedure for total nucleic extraction (individual extraction-only one spot in each extraction tube):**

- Use a standard 6mm-diameter hole puncher to cut a whole spot (approximately 50 µL blood in a single spot) into individual tubes.
- Add **900 µL** of the lysis buffer
- Incubate the tubes at 65°C and 250 rpm shaking speed for 2.5 hours in an orbital shaker. Note: different kind of tube rack can hold the tubes in different ways. Check that lysis buffer has mixed with blood homogenously. If it is seen that blood is more concentrated at the bottom of the tube and a clearer solution at the top, shaking speed might need to increase. Similarly, if you see lysate at lid of the tube, then reduce the speed of shaking.
- After incubation centrifuge the tubes at 13000rpm speed for 15 second.
- Set up the HiBind® DNA mini columns (Omega Bio-tek, Norcross, GA) on the 2mL collection tube
- Transfer 700µL of the supernatant into the DNA column
- Centrifuge the column at 2000rpm for 2 minutes
- Then centrifuge the column at 8000rpm for 1 minutes
- Discard the flow through in a bottle and dry the top of the collection tube by inverting and pressing on a layer of paper towel. Hold the column in another while discarding the liquid and drying the collection tube.
- Re-set up the column on the collection tube
- Add 500 µL of the “wash buffer 1” in the column
- Centrifuge at 8000rpm for one minute.
- Again, discard the flow through in a bottle and dry the top of the collection tube as done in the previous step
- Re-set up the column on the collection tube
- Add 500 µL of the “wash buffer 2” in the column
- Centrifuge at 13000 rpm for three minutes.
- Discard the collection tube
- Place the column on the top of 1.5mL micro-centrifuge tube
- Add 50 µL TE buffer in the middle of the white material, no buffer should be seen at the body of the column
- Wait for 10 minutes
- Centrifuge at 8000rpm for one minute.
- Discard the column and collect total nucleic acid in the 1.5mL micro-centrifuge tube
- Make 3-4 aliquots and keep at -80C until use it.

**Step by step procedure for total nucleic extraction (batch extraction- total 4 spots in a single extraction tube):**

- Use a standard 6mm-diameter hole puncher to cut up to 4 whole spots (approximately 50 µL blood in a single spot) into the single tubes.
- Add **1300 µL** of the lysis buffer
- Incubate the tubes at 65°C and 250 rpm shaking speed for 2.5 hours in an orbital shaker. Note: different kind of tube rack can hold the tubes in different ways. Check that lysis buffer has mixed with blood homogenously. If it is seen that blood is more concentrated at the bottom of the tube and a clearer solution at the top, shaking speed might need to increase. If the lysate comes to the lid of the tubes, reduce the speed.
- After incubation centrifuge the tubes at 13000rpm speed for 15 second.
- Set up the HiBind® DNA mini columns (Omega Bio-tek, Norcross, GA) on the 2mL collection tube
- Transfer 700µL of the supernatant into the DNA column
- Centrifuge the column at 3000rpm for 2 minutes
- Then centrifuge the column at 8000rpm for 1 minutes
- Discard the flow through in a bottle and dry the top of the collection tube by inverting and pressing on a layer of paper towel. Hold the column in another while discarding the liquid and drying the collection tube.
- Re-set up the column on the collection tube
- Add 500 µL of the “wash buffer 1” in the column
- Centrifuge at 8000rpm for one minute.
- Again, discard the flow through in a bottle and dry the top of the collection tube as done in the previous step
- Re-set up the column on the collection tube
- Add 500 µL of the “wash buffer 2” in the column
- Centrifuge at 13000 rpm for three minutes.
- Discard the collection tube
- Place the column on the top of 1.5mL micro-centrifuge tube
- Add 50 µL TE buffer in the middle of the white material, no buffer should be seen at the body of the column
- Wait for 10 minutes
- Centrifuge at 8000rpm for one minute.
- Discard the column and collect total nucleic acid in the 1.5mL micro-centrifuge tube
- Make 3-4 aliquots and keep at -80C until use it.

**Waste disposal:**

The cell lysate and flow through after first wash should be kept in a bottle. Once the bottle is full, it can be requested to pick up through chematix as a bottle of contaminated material. It has blood contaminated stuff as well as 2-marceptoethanol. Tubes and tips can be discarded with regular biohazard.

**Acknowledgement:** The original protocol was provided by Chris Plowe’s laboratory at the University of Maryland

**Reference:**

Zainabadi K, et al. A novel method for extracting nucleic acids from dried blood spots for ultrasensitive detection of low-density Plasmodium falciparum and Plasmodium vivax infections. *Malaria journal* 2017, **16**:377.
