# Supplementary material for: Ultrasensitive loop mediated isothermal amplification (US-LAMP) to detect malaria for elimination
Source: Malar J. 2019 Oct 16;18:350. doi: 10.1186/s12936-019-2979-4 (PMC6796404; doi:10.1186/s12936-019-2979-4)
Supplement: Supplementary file 4 — Additional file 4. Reverse transcriptase Real Time PCR protocol for dried blood spot extracted samples. [file 12936_2019_2979_MOESM4_ESM.docx]

Hazards associated with equipment/machinery/materials/technique/process

- Hazardous in case of skin contact, of eye contact, of inhalation or ingestion.

Personal protective equipment

- Personnel handling chemical must wear proper personal protective equipment; laboratory coat, nitrile gloves and closed-toe shoes

Emergency procedures

- Users must be familiar with the Emergency response procedures, Use of Emergency eye wash/ shower station, Spill Response Procedure and use of the Spill response kit.

Reagents:

- Primers and probes are synthesized from Sigma-aldrich and Thermofisher Scientific.
- QuantiTect Multiplex RT PCR kit (Cat: 204643)- Qiagen incorporation

**Primer sequences: (Actin gene as the internal control)**

Fal.TM.FOR: 5'-CCGACTAGGTGTTGGATGAAAGTGTTAA

Plas.TM.REV: 5'-AACCCAAAGACTTTGATTTCTCATAA -3'

Viv.TM.FOR: 5’-CCGACTAGGCTTTGGATGAAAGATTTTA-3’

Actin_Ultra_F: 5'-ACCGAGCGCGGCTACAG-3'

Actin_Ultra_R: 5'-CTTAATGTCACGCACGATTTCC-3'

**Probes:**

Pf_Ultra_CB: Cy5/agcaatctaaaagtcacctcgaaagatgact/3BHQ_2

Pv_Ultra_FB: FAM/agcaatctaagaataaactccgaagagaaaattct/3BHQ_1

Actin_Ultra_P: VIC-TTCACC ACCACGGCCGAGC-MGB-3'

**Composition of the master mix (one reaction):**

2X QuatiTect RT PCR master mix = 5.0µL

10 µM Fal.TM.FOR: = 0.45 µL

10 µM Plas.TM.REV: = 0.6 µL

10 µM Viv.TM.FOR: =0.1 µL

10 µM Actin_Ultra_F: =0.1 µL

10 µM Actin_Ultra_R: =0.1 µL

10 µM Pf_Ultra_CB: =0.1 µL

10 µM Pv_Ultra_FB: =0.1 µL

10 µM Actin_Ultra_P: =0.1 µL

QuantiTect RT mix: =0.1 µL

Nuclease free warer =1.75 µL

Template =1.5 µL

Total =10 µL

**PCR Cycles:**

50°C for 20 minutes

95°C for 15 minutes

94°C 45 seconds

60°C for 75 seconds (add a plate read step here)

Repeat step 3-4 for 45 cycles

# Procedure:

**Assay preparation**

- Use information on the above for setting up PCR reactions and the CFX 96 Real Time system cycling conditions
- Determine the number of samples (n) you need to amplify.
- Calculate the volume of master mix to make necessary amount of master mix according to the above composition
- Note: Prepare master mix for 2-5 extra samples (eg: n+5) to allow enough volume for pipetting.
- Add 8.5 µL per well of master mix to the Biorad 96 well microplate (Cat: HSL9641) or individual low-profile tubes
- Add 1.5µL of the prepared standards in the wells selected for corresponding concentrations
- Add 1.5 µL of positive control nucleic acid to one well, 1.5µL negative control in another well, and 1.5 µL of water to another well as No Template Controls (NTC).
- Add 1.5 µL of extracted nucleic acid sample to appropriate wells.
- Note: It is recommended that test DNA samples also be added in duplicate wells, especially if the parasite DNA concentration in the sample is expected to be low.
- Carefully and thoroughly seal the plate with a Biorad optical adhesive seal (Cat: MSB1001) and briefly centrifuge the plate if possible, then place it in the CFX Real Time PCR System.
- If individual tubes are used, close the lead tightly and spin down the liquids.
- Set up the PCR cycling conditions on the CFX96 Real Time System and start the PCR reaction
- Machine automatically close the lead and run will start, and the data will be saved in the data file.

**Data analysis:**

- Check actin gene amplification, if it is not amplified in a particular well, the assay is invalid for that well not for the whole plate, indicates extraction problem.
- Fix the threshold bars at RFU=100 for Cy5 and FAM
- Then you will get Ct value for individual type of detection
- **A conservative maximum cycle cutoff of 35 is advisable to prevent low-level false positives**
- A standard curve will be generated automatically by the machine which will include curve fitness, Ct value for individual standards. Curve is created by plotting Ct value against Log of Parasite concentration/mL.
- If the curve fitness looks good, use it for quantitation
- Select “data analysis”
- Machine will auto generate Ct value for individual wells as well as individual fluorophore. Additionally, software will calculate parasite concentration for individual fluorophore. **That is your parasite/mL in a sample.**

**Standard curve:**

- Prepare the standards for Pf and Pv. Start with 10000, 1000, 100, 50, 25, 10 parasites/mL concentrations. Any microscopy counted sample can be diluted with uninfected donor blood to have these concentrations^*^
- Once dilutions are ready, spot 50µL on to Whatmann 903 protein saver card
- Dry the spots overnight
- Extract the total Nucleic acid from those spots according to “Supplementary protocol-1”
- Label with concentrations
- Load 1.5 µL in well containing the master mix

# Safety and Precautions

- Use proper lab etiquette when working in the laboratory. Wear gloves, labcoat and safety glasses when handling specimens.
- Ensure that all equipment (pipettes, centrifuges, water baths, etc.) is properly calibrated and DNase/RNase-free prior to use.
- Take care when applying or removing plate sealant, to prevent cross-contamination of adjacent wells.
- Take utmost care during pipetting steps to prevent cross contamination of samples or PCR products.
- Use an ice bath to hold the PCR master mix and/or PCR reagents during PCR master mix preparation.
- Do not vortex the PCR mix. Mix gently by tapping the side of the tube containing the master mix or mix by pipetting. Use light centrifugation to collect all liquid to the bottom of the PCR plate or tube.
- Store extracted or amplified DNA at -80 °C (2-8 °C storage is allowed for ≤ 24 hours; -20 °C storage is allowed for ≤ 2 weeks).
- Do not thaw the RNA at room temperature, always keep in a ice bucket

**Waste disposal procedures**

- liquid and solid waste must be disposed of according to the University of Calgary Hazardous Material Disposal Manual procedures through Chematix.

**Maintenance**

- Take the plate or tubes from the instrument and turn of the software first. Then turn on the off the machine and turn off the electrical switch. Follow the reverse sequence to turn on the machine.

**Acknowledgement:** The original protocol was provided by Chris Plowe’s laboratory at the University of Maryland.

**Reference:**

Adams, M. *et al*. An ultrasensitive reverse transcription polymerase chain reaction assay to detect asymptomatic low-density *Plasmodium falciparum* and *Plasmodium vivax* infections in small volume blood samples. Malaria Journal 2015, **14**: 520

Kamau, E. *et al*. Development of a Highly Sensitive Genus-Specific Quantitative Reverse Transcriptase Real-Time PCR Assay for Detection and Quantitation of Plasmodium by Amplifying RNA and DNA of the 18S rRNA Genes. Journal of Clinical Microbiology, Aug. 2011, p. 2946–2953

Zainabadi K, et al. A novel method for extracting nucleic acids from dried blood spots for ultrasensitive detection of low-density Plasmodium falciparum and Plasmodium vivax infections. *Malaria journal* 2017, **16**:377.
